# A Regularization Homotopy Strategy for the Constrained Parameter Inversion of Partial Differential Equations

**DOI:** 10.3390/e23111480

**Published:** 2021-11-09

**Authors:** Tao Liu, Runqi Xue, Chao Liu, Yunfei Qi

**Affiliations:** 1 School of Mathematics and Statistics, Northeastern University at Qinhuangdao, Qinhuangdao 066000, China; math_rqxue123@163.com (R.X.); liuchao@mail.neu.edu.cn (C.L.); 2 Eighth Geological Bridage of Hebei Geology, Mineral Resources Exploration Bureau, Qinhuangdao 066000, China; geo_yfqi@163.com

**Keywords:** parameter inversion, partial differential equation, Tikhonov regularization, homotopy method, constraints

## Abstract

The main difficulty posed by the parameter inversion of partial differential equations lies in the presence of numerous local minima in the cost function. Inversion fails to converge to the global minimum point unless the initial estimate is close to the exact solution. Constraints can improve the convergence of the method, but ordinary iterative methods will still become trapped in local minima if the initial guess is far away from the exact solution. In order to overcome this drawback fully, this paper designs a homotopy strategy that makes natural use of constraints. Furthermore, due to the ill-posedness of inverse problem, the standard Tikhonov regularization is incorporated. The efficiency of the method is illustrated by solving the coefficient inversion of the saturation equation in the two-phase porous media.

## 1. Introduction

Inverse problems of partial differential equations arise in a variety of practical problems in science and engineering. These range from biomedical and geophysical imaging to groundwater flow modeling [1,2,3,4,5,6,7,8,9]. It is very significant to conduct research into the theory of inverse problems and their applications. Generally, it is very difficult to solve inverse problems. This is due in part to the ill-posedness of the problem itself, which causes the solution not to depend continuously on the measurement data. It is also due to the presence of numerous local minima in the cost function, which hinders the convergence of the ordinary numerical methods. In order to overcome these two difficulties, a homotopy strategy that makes natural use of constraints is designed for the general parameter inversion of partial differential equations.

The homotopy method is a novel and effective method which has been successfully applied to find solutions of various nonlinear problems such as zeros and fixed points of mappings and so on. A remarkable advantage of this method is that the algorithm generated by it is globally convergent under certain weak assumptions [10]. Recently, it has also been extended to dealing with inverse problems. Keller and Perozzi [11] may be the first researchers to have used the homotopy method to solve the inverse problems. Then, Vasco [12] combined a homotopy path-following formalism with the singularity theory to track multiple solutions of a geophysical inverse problem. Shidfar et al. [13] used a weighted homotopy analysis method to solve the inverse problem of identifying an unknown source term in a parabolic equation. Zhao et al. [14] developed an adaptive homotopy method for the parameter identification inverse problem of a nonlinear diffusion equation. Hu et al. [15] constructed a homotopy approach to improve the PEM identification of ARMAX models. Cao and Han [16] presented the convergence analysis of the homotopy perturbation method for solving nonlinear ill-posed operator equations.

Generally, a parameter inversion identifies parameters using only measurement data recorded on the surface of the object to be measured. However, these measurement data may have a low signal-to-noise ratio. To suppress the noise and improve the quality of inversion, constraints have been widely used in the inversion fields, such as remote sensing of the environment [17,18], atmospheric research [19,20], geological exploration [21,22], petrophysics [23,24] and so on. The main reason for this is that the constraint data, which are recorded from the interior of the object to be measured, may be less noisy than the surface data.

In this paper, we study the parameter inversion problem of partial differential equations considering not only the measurement data but also the constraint data. Firstly, the constrained parameter inversion problem is formulated as the constrained minimization problem; then, this constrained minimization problem is transformed into an unconstrained minimization problem by the use of the penalty function method. Secondly, the homotopy method is introduced and combined with Tikhonov regularization, a successive approximation method, to give a widely convergent inversion strategy. In general terms, the successive approximation method aims to reduce uncertainty, which could be measured as decreasing the entropy of the distribution of uncertain system parameters, at a computational cost that is analogous to energy supply. We call this method the regularization homotopy strategy. Finally, as a practical application, a distributed parameter inversion problem of the saturation equation in the two-phase porous media is solved.

The contents of this paper are outlined as follows. The constrained parameter inversion problem of partial differential equations is described in Section 2. A brief introduction of homotopy theory is given in Section 3. The regularization homotopy strategy is presented in Section 4. In order to test the method’s effectiveness, numerical simulations are carried out for the parameter inversion of the saturation equation in the fractional flow formulation of the two-phase porous media flow equations in Section 5. Finally, in Section 6, some conclusions are given.

## 2. Inversion Model

Consider the following partial differential equation

(1) L(p(x),t)u(x,t)=0,x∈Ω,t>0,

where $x={\left(x_{1},x_{2},\ldots ,x_{n}\right)}^{⊤}$, $\Omega \subset R^{n}$ is a bounded domain, u(x,t) is a sufficiently smooth function defined on Ω×(0,∞), and *L* is the differential operator. The initial and boundary conditions are

(2) Iu(x,0)=φ(x),x∈Ω,

(3) Bu(x,t)=ϕ(x,t),x∈∂Ω,t>0,

where ∂Ω is the boundary of Ω, and *I* and *B* are the initial condition operator and boundary condition operator, respectively. If p(x), φ(x), ϕ(x,t) are known, Equations (1)–(3) form the direct problem to determine u(x,t).

If p(x) is unknown, given the additional condition

(4) Au(x,t)=ψ(x,t),x∈Γ⊂Ω,t>0,

with Γ being a part of Ω and *A* the additional condition operator, Equations (1)–(4) form the general parameter inversion of partial differential equations.

In the practical problems, the parameter p(x) is usually in the discrete form. Let

$$P^{⊤}=\left(p_{1},p_{2},\ldots ,p_{Q}\right)$$

denote the parameters to be determined in the discrete form. As the solution u(x,t) of Equations (1)–(3) nonlinearly depends on p(x), a nonlinear operator K(P) can be defined as

K(P)=Au(P;x,t)−ψ(x,t)x∈Γ⊂Ω,t>0,

then, the parameter inversion is reduced to the output least squares problem:

$${\min∥K\left(P\right)∥}^{2}.$$

Denote the admissible set

$$\Sigma =\{P:p_{i_{1}}={\hat{p}}_{i_{1}},p_{i_{2}}={\hat{p}}_{i_{2}},\ldots ,p_{i_{d}}={\hat{p}}_{i_{d}}\},$$

where ${\hat{p}}_{i_{1}},{\hat{p}}_{i_{2}},\ldots ,{\hat{p}}_{i_{d}}$ are obtained from the constraint data, and $1\leq i_{1}<i_{2}<\cdots <i_{d}\leq Q$. Then, the constrained parameter inversion can be transformed into the solution of the problem

(5) $$\min_{P\in \Sigma }{∥K\left(P\right)∥}^{2}.$$

Let

$${\hat{P}}^{⊤}=\left({\hat{p}}_{i_{1}},{\hat{p}}_{i_{2}},\ldots ,{\hat{p}}_{i_{d}}\right),$$

$${\left(EP\right)}^{⊤}=\left(p_{i_{1}},p_{i_{2}},\ldots ,p_{i_{d}}\right),$$

where *E* is the extraction operator. It is obvious that $∥EP-\hat{P}∥=0$ holds for P∈Σ. Thus, the least squares problem (5) can be written, without constraint, as 

(6) $$\min\{∥K\left(P\right){∥}^{2}+\alpha ∥EP-\hat{P}{∥}^{2}\},$$

where α is a constraint parameter to determine the strength of the constraint. The solutions of Equations (5) and (6) are close to each other when α is large enough. Therefore, in order for solution of Equation (6) to approximate the solution of Equation (5) well, it is necessary that α is specified as large enough in the specific inversion process.

## 3. Homotopy Theory

A brief introduction of the homotopy theory, which is applicable to nonlinear operator equations such as K(P)=0, is given in this section. For more details, interested readers are referred to the tutorial provided by Watson [10].

By adding a homotopy parameter κ∈[0,1] and an artificially constructed function C(P) to the “target function” K(P), a homotopy map F(P,κ) is constructed so that

F(P,0)=C(P),F(P,1)=K(P),

where C(P) is usually chosen as a simple function, and we assume that the solution of C(P)=0 is known. For example, we may choose $C\left(P\right)=P-P^{0}$, and the known solution of C(P)=0 is $P^{0}$. $P^{0}$ can be chosen as the initial value of the whole computation. It is assumed that there is a curve P=P(κ), κ∈[0,1] satisfying the homotopy equation

(7) F(P,κ)=0.

Obviously, $P\left(0\right)=P^{0}$, $P\left(1\right)=P^{*}$, and $P^{*}$ is the solution of K(P)=0. Thus, P(κ) is a continuous curve connecting $P^{0}$ with $P^{*}$. If some numerical method is applied to trace this curve, the solution $P^{*}$ will eventually be reached.

Generally, there are two ways to trace the curve P(κ). The first method is on the basis of the hypothesis that F(P,κ) is differentiable with respect to *P* and κ. By differentiating each side of Equation (7),

(8) $$F_{P}^{\prime }\left(P,\kappa \right)dP+F_{\kappa }^{\prime }\left(P,\kappa \right)d\kappa =0.$$

Because *P* satisfies the initial condition $P\left(0\right)=P^{0}$, the problem of determining P(κ) is transformed into an initial value problem of ordinary differential Equation (8). An approximation of $P^{*}=P\left(1\right)$ can be obtained by using numerical integration. The second method is first to divide the interval [0,1] into $0=\kappa_{0}<\kappa_{1}<\cdots <\kappa_{S}=1$ and then to solve the operator equations sequentially:

(9) $$F(P,\kappa_{s})=0,\;s=1,2,\ldots ,S.$$

By induction, the solution $P^{s}$ of the *s*th equation is known, so $P^{s}$ can be used as the initial approximation of the (s+1)th equation of Equation (9). $P^{s}$ is such a good approximation of $P^{s+1}$ that we only use a locally convergent method to obtain $P^{s+1}$, as long as $\kappa_{s+1}-\kappa_{s}$ is small enough. In this article, the latter method is used as our path tracing method.

## 4. Homotopy Strategy

It is well known that the inverse problem in Equation (6) is ill-posed, so the regularization method must be employed:

(10) $$\min\{∥K\left(P\right){∥}^{2}+\alpha ∥EP-\hat{P}{∥}^{2}+\beta ∥P-P^{0}{∥}^{2}\},$$

where $P^{0}$ and β are the initial estimate and regularization parameter, respectively.

Obviously, Equation (10) is equivalent to the corresponding normal equation

(11) $$K^{\prime }{\left(P\right)}^{*}K\left(P\right)+\alpha E^{*}\left(EP-\hat{P}\right)+\beta \left(P-P^{0}\right)=0,$$

where * denotes the adjoint operator. For the sake of avoiding the impact of the second derivative, a successive linearization method is used to construct a basic iterative method. It is also interesting to use other methods, such as gradient [25], gradientless [26], minimization with constraints [27] and stochastic approaches [28].

Assume that the *k*th approximation $P^{k}$ of Equation (11) has been found. To compute the next approximation $P^{k+1}$, the linear function $L_{k}\left(P\right)=K^{\prime }\left(P^{k}\right)\left(P-P^{k}\right)+K\left(P^{k}\right)$ is used instead of K(P), and the cost function in Equation (10) is replaced by

$$∥L_{k}{\left(P\right)∥}^{2}+\alpha ∥EP-\hat{P}{∥}^{2}+\beta {∥P-P^{0}∥}^{2}.$$

Its corresponding normal equation is

$$K^{\prime }{\left(P^{k}\right)}^{*}\left(K^{\prime }\left(P^{k}\right)\left(P-P^{k}\right)+K\left(P^{k}\right)\right)+\alpha E^{*}\left(EP-\hat{P}\right)+\beta \left(P-P^{0}\right)=0,$$

the solution of which is exactly the next approximation $P^{k+1}$; that is,

(12) $$\begin{array}{cc} \; & P^{k+1}=P^{k}-{\left[K^{\prime }{\left(P^{k}\right)}^{*}K^{\prime }\left(P^{k}\right)+\alpha E^{*}E+\beta I\right]}^{-1}\\ \; & \times [K^{\prime }{\left(P^{k}\right)}^{*}K\left(P^{k}\right)+\alpha E^{*}\left(EP^{k}-\hat{P}\right)+\beta \left(P^{k}-P^{0}\right)],\;k=0,1,2,\ldots \end{array}$$

This iterative method has a fast convergence speed and good stability; however, it only has local convergence. As a matter of fact, Equation (12) is a variant of the iteratively regularized Gauss–Newton method [29] and has the same computational cost as the latter.

In an attempt to overcome the local convergence problem, the homotopy method is introduced to solve Equation (11). Consider the following fixed-point homotopy equation

(13) $$F\left(P,\kappa \right)=\kappa \left[K^{\prime }{\left(P\right)}^{*}K\left(P\right)+\alpha E^{*}\left(EP-\hat{P}\right)+\beta \left(P-P^{0}\right)\right]+\left(1-\kappa \right)\left(P-P^{0}\right)=0,$$

where κ∈[0,1] is the homotopy parameter. In addition, Equation (13) can be rearranged as

(14) $$\kappa \left[K^{\prime }{\left(P\right)}^{*}K\left(P\right)+\alpha E^{*}\left(EP-\hat{P}\right)\right]+\left(1-\kappa +\kappa \beta \right)\left(P-P^{0}\right)=0.$$

With the purpose of finding the solution of Equation (11), the interval [0,1] is divided into $0=\kappa_{0}<\kappa_{1}<\cdots <\kappa_{S}=1$, and for $\kappa =\kappa_{s}$, the above basic iterative method is used to solve Equation (14) sequentially. The known solution $P^{0}$ of $F(P,\kappa_{0})$ can be regarded as the initial estimate of the next equation $F(P,\kappa_{1})$. Suppose that the solution $P^{s}$ of $F(P,\kappa_{s})$ has been found; the successive linearization method, which is performed with Equation (12), can be applied to $F(P,\kappa_{s+1})$. Therefore, we can construct the iterative formula as follows:

(15) $$\begin{array}{cc} \; & P_{k+1}^{s}=P_{k}^{s}-[\kappa_{s}K^{\prime }{\left(P_{k}^{s}\right)}^{*}K^{\prime }\left(P_{k}^{s}\right)+\kappa_{s}\alpha E^{*}E\\ \; & +\left(1-\kappa_{s}+\kappa_{s}\beta \right)I{]}^{-1}\times [\kappa_{s}K^{\prime }{\left(P_{k}^{s}\right)}^{*}K\left(P_{k}^{s}\right)+\kappa_{s}\alpha E^{*}\left(EP_{k}^{s}-\hat{P}\right)\\ \; & +\left(1-\kappa_{s}+\kappa_{s}\beta \right)\left(P_{k}^{s}-P^{0}\right)],\;k=0,1,\ldots ,s_{T},\\ \; & P_{0}^{s}=P^{s-1},\;P^{s}=P_{s_{T}+1}^{s},\;s=1,2,\ldots ,S. \end{array}$$

In reality, the final iterative result $P^{S}$ of Equation (15), which is the solution of F(P,1), is none other than the regularized solution of Equation (11). This regularization homotopy algorithm is globally convergent to solve the constrained parameter inversion of partial differential equations. For the global convergence analysis of the homotopy method, see [16,30].

Let $\kappa_{s}=\frac{s}{S}$ and $s_{T}\equiv 0$; then, we have a simplified version of Equation (15):

(16) $$\begin{array}{cc} \; & P^{s+1}=P^{s}-[\frac{s}{S}K^{\prime }{\left(P^{s}\right)}^{*}K^{\prime }\left(P^{s}\right)+\frac{s}{S}\alpha E^{*}E\\ \; & +\left(1-\frac{s}{S}+\frac{s}{S}\beta \right)I{]}^{-1}\times [\frac{s}{S}K^{\prime }{\left(P^{s}\right)}^{*}K\left(P^{s}\right)+\frac{s}{S}\alpha E^{*}\left(EP^{s}-\hat{P}\right)\\ \; & +\left(1-\frac{s}{S}+\frac{s}{S}\beta \right)\left(P^{s}-P^{0}\right)],\;s=0,1,\ldots ,S-1. \end{array}$$

**Figure 1:**
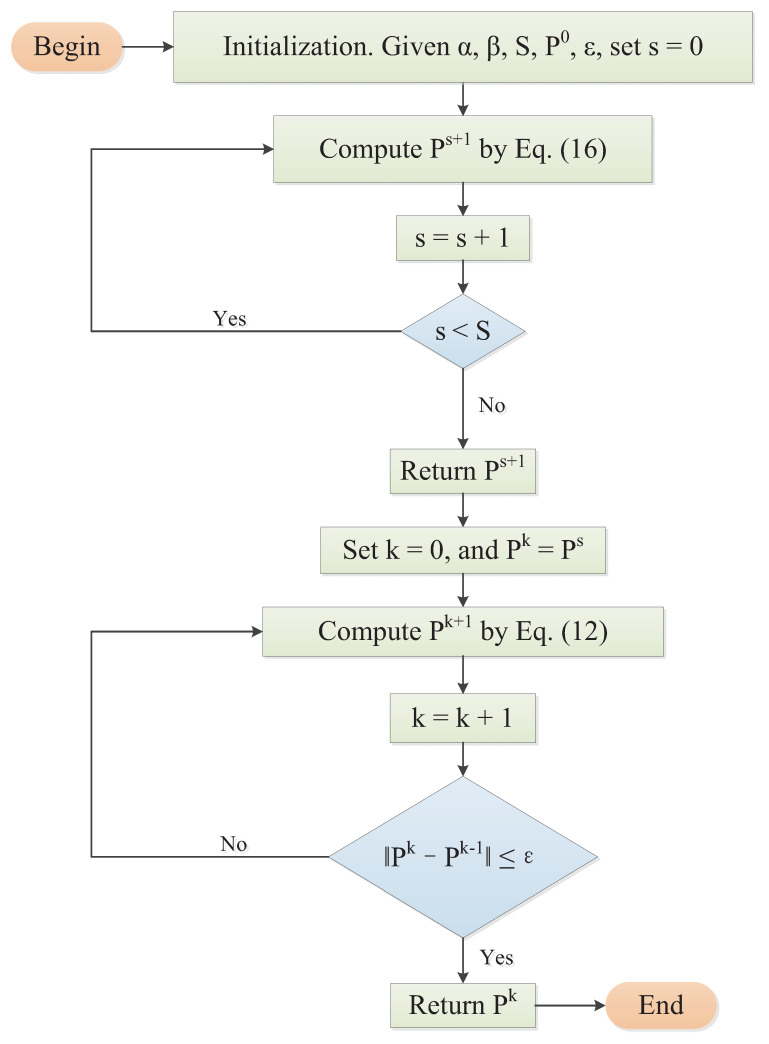
Flowchart of homotopy method with constraints.

The iterative result $P^{S}$ of Equation (16) can be used as the initial estimate of Equation (12). Then, iteration using Equation (12) is repeated until the regularized solution of Equation (11) is found. Consequently, Equations (12) and (16) can form a stable, globally convergent and fast method, as described in Algorithm 1. The flowchart of Algorithm 1 is shown in Figure 1.
**Algorithm 1:** Homotopy method with constraints.1. Initialization. Given *α*, *β*, *S*, $P^{0}$, *ε*, s=0.2. Compute $P^{s+1}$ by Equation (16).3. Let s=s+1 and check if s<S then go to Step 2, else if s≥S, then return $P^{s}$.4. set k=0 and $P^{k}=P^{s}$.5. Compute $P^{k+1}$ by Equation (12).6. Let k=k+1 and check if $∥P^{k}-P^{k-1}∥>\varepsilon$, then go to Step 5, else if $∥P^{k}-P^{k-1}∥\leq \varepsilon$, then return $P^{k}$.

If α=0, Equation (10) is simply the ordinary parameter inversion of partial differential equations, and Equations (12) and (16) can respectively be rewritten as follows:

(17) $$\begin{array}{cc} \; & P^{s+1}=P^{s}-{\left[\frac{s}{S}K^{\prime }{\left(P^{s}\right)}^{*}K^{\prime }\left(P^{s}\right)+\left(1-\frac{s}{S}+\frac{s}{S}\beta \right)I\right]}^{-1}\\ \; & \times [\frac{s}{S}K^{\prime }{\left(P^{s}\right)}^{*}K\left(P^{s}\right)+\left(1-\frac{s}{S}+\frac{s}{S}\beta \right)\left(P^{s}-P^{0}\right)],\\ \; & s=0,1,\ldots ,S-1, \end{array}$$

and

(18) $$\begin{array}{cc} \; & P^{k+1}=P^{k}-{\left[K^{\prime }{\left(P^{k}\right)}^{*}K^{\prime }\left(P^{k}\right)+\beta I\right]}^{-1}\\ \; & \times [K^{\prime }{\left(P^{k}\right)}^{*}K\left(P^{k}\right)+\beta \left(P^{k}-P^{0}\right)],\;k=0,1,2,\ldots \end{array}$$

After the comparison of the proposed method and the other well-known iterative methods (such as the regularized Gauss–Newton method), it is found that their calculation amounts and storage requirements are the same at each step. The application of the first-order derivative, the evaluation of the adjoint operator and the forward-modeling run are of primary importance; the most important factor is, however, that the convergence domain of the proposed method is much wider than the other iterative methods, such as the regularized Gauss–Newton method.

## 5. An Application

### 5.1. Mathematical Model

This section studies the following permeability parameter inversion of the nonlinear convection–diffusion equation, which can be considered as the saturation equation in the fractional flow formulation of the two-phase porous media flow equations

(19) $$\left\{\begin{array}{c} u_{t}+\frac{\partial }{\partial x}f\left(u\right)+\frac{\partial }{\partial y}h\left(u\right)-\nabla \cdot \left(p\left(x,y\right)N\left(u\right)\nabla u\right)=s\left(x,y,t\right),\;\left(x,y\right)\in \Omega ,\;t>0,\\ u(x,y,0)=\varphi (x,y),\;(x,y)\in \Omega ,\\ u(x,y,t)=\phi (x,y,t),\;(x,y)\in \partial \Omega ,\;t>0,\\ u\left(x^{g},y^{g},t\right)=\psi \left(x^{g},y^{g},t\right),\;g=1,2,\ldots ,G,\;t>0. \end{array}\right.$$

Let Ω be the unit square, and after the finite difference discretization, Equation (19) turns into

(20) $$\left\{\begin{array}{c} \frac{u_{i,j}^{m}-u_{i,j}^{m-1}}{\Delta t}+\nabla \cdot \left(f\left(u_{i,j}^{m-1}\right),h\left(u_{i,j}^{m-1}\right)\right)-\nabla \cdot \left(p_{i,j}N_{i,j}^{m}\nabla u_{i,j}^{m}\right)=s\left(i\Delta x,j\Delta y,m\Delta t\right),\\ i=1,2,\ldots ,n_{1}-1;\;j=1,2,\ldots ,n_{2}-1,\\ u_{i,j}^{0}=\varphi \left(i\Delta x,j\Delta y\right),\;i=0,1,\ldots ,n_{1};\;j=0,1,\ldots ,n_{2},\\ u_{0,j}^{m}=\phi \left(0,j\Delta y,m\Delta t\right),\;j=0,1,\ldots ,n_{2},\\ u_{1,j}^{m}=\phi \left(1,j\Delta y,m\Delta t\right),\;j=0,1,\ldots ,n_{2},\\ u_{i,0}^{m}=\phi \left(i\Delta x,0,m\Delta t\right),\;i=0,1,\ldots ,n_{1},\\ u_{i,1}^{m}=\phi \left(i\Delta x,1,m\Delta t\right),\;i=0,1,\ldots ,n_{1},\\ u_{x^{g},y^{g}}^{m}=\psi \left(x^{g},y^{g},m\Delta t\right),\;g=1,2,\ldots ,G, \end{array}\right.$$

where Δx, Δy are the step sizes of the rectangular grid in the *x* and y-directions, respectively, Δt is the time step size, $n_{1}=\frac{1}{\Delta x}$, $n_{2}=\frac{1}{\Delta y}$, $u_{i,j}^{m}=u\left(i\Delta x,j\Delta y,m\Delta t\right)$, $p_{i,j}=p\left(i\Delta x,j\Delta y\right)$. $\nabla \cdot (f\left(u_{i,j}^{m-1}\right),h\left(u_{i,j}^{m-1}\right))$ and $\nabla \cdot (p_{i,j}N_{i,j}^{m}\nabla u_{i,j}^{m})$ are the discretization expressions of the convection term and the diffusion term, respectively. Equation (20) can define a vector-valued function H:P→U, where

$$P^{⊤}=\left(p_{1,1},p_{1,2},\ldots ,p_{1,n_{2}},p_{2,1},p_{2,2},\ldots ,p_{2,n_{2}},\ldots ,p_{n_{1},1},p_{n_{1},2},\ldots ,p_{n_{1},n_{2}}\right),$$

$$\begin{array}{cc} & U^{⊤}=(u_{x^{1},y^{1}}^{1},u_{x^{2},y^{2}}^{1},\ldots ,u_{x^{G},y^{G}}^{1},u_{x^{1},y^{1}}^{2},u_{x^{2},y^{2}}^{2},\ldots ,u_{x^{G},y^{G}}^{2},\\ & \ldots ,u_{x^{1},y^{1}}^{M},u_{x^{2},y^{2}}^{M},\ldots ,u_{x^{G},y^{G}}^{M}). \end{array}$$

Let $\psi_{x^{g},y^{g}}^{m}=\psi \left(x^{g},y^{g},m\Delta t\right)$; then, the measurement data form the vector

$$\begin{array}{cc} & \Psi^{⊤}=(\psi_{x^{1},y^{1}}^{1},\psi_{x^{2},y^{2}}^{1},\ldots ,\psi_{x^{G},y^{G}}^{1},\psi_{x^{1},y^{1}}^{2},\psi_{x^{2},y^{2}}^{2},\ldots ,\psi_{x^{G},y^{G}}^{2},\\ & \ldots ,\psi_{x^{1},y^{1}}^{M},\psi_{x^{2},y^{2}}^{M},\ldots ,\psi_{x^{G},y^{G}}^{M}). \end{array}$$

Therefore, the nonlinear operator K(P)=H(P)−Ψ.

Let

$${\hat{P}}^{⊤}=\left({\hat{p}}_{x_{0},1},{\hat{p}}_{x_{0},2},\ldots ,{\hat{p}}_{x_{0},n_{2}}\right),$$

where $\hat{P}$ is the parameter from the well logs of a well located at point $x_{0}$. Subsequently, the constrained parameter inversion of the nonlinear convection-diffusion equation turns into

$$\min\{∥H\left(P\right)-\Psi {∥}^{2}+\alpha ∥EP-\hat{P}{∥}^{2}\}.$$

where

$$E={\left(\begin{array}{cccccccccccc} 0 & 0 & \ldots & 0 & 1 & 0 & \ldots & 0 & 0 & 0 & \ldots & 0\\ 0 & 0 & \ldots & 0 & 0 & 1 & \ldots & 0 & 0 & 0 & \ldots & 0\\ \vdots & \vdots & & \vdots & \vdots & \vdots & \ddots & \vdots & \vdots & \vdots & & \vdots \\ 0 & 0 & \ldots & 0 & 0 & 0 & \ldots & 1 & 0 & 0 & \ldots & 0 \end{array}\right)}_{n_{2}\times \left(n_{1}\times n_{2}\right)}$$

is the matrix such that ${\left(EP\right)}^{⊤}=\left(p_{x_{0},1},p_{x_{0},2},\ldots ,p_{x_{0},n_{2}}\right)$.

### 5.2. Numerical Simulations

To show the feasibility of the proposed method and illustrate its numerical behavior, the numerical simulation procedure is carried out for two concrete models in MATLAB R2018a environment. We choose

$$\begin{array}{cc} & f\left(u\right)=\frac{u^{2}\left(1-5\left(1-u^{2}\right)\right)}{u^{2}+{\left(1-u\right)}^{2}},\;h\left(u\right)=\frac{u^{2}}{u^{2}+{\left(1-u\right)}^{2}},\;s\left(x,y,t\right)=0,\\ & N\left(u\right)=u^{2}-u+1,\;\varphi \left(x,y\right)=\sin\left(\pi x\right)\sin\left(\pi y\right),\;\phi \left(x,y,t\right)=0,\\ & \Delta x=\Delta y=1/24,\;\Delta t=0.002,\;x_{0}=12/24,\\ & \alpha =10^{4},\;\beta =10^{-5},\;S=10,\;P^{0}\equiv 5. \end{array}$$

**Figure 2:**
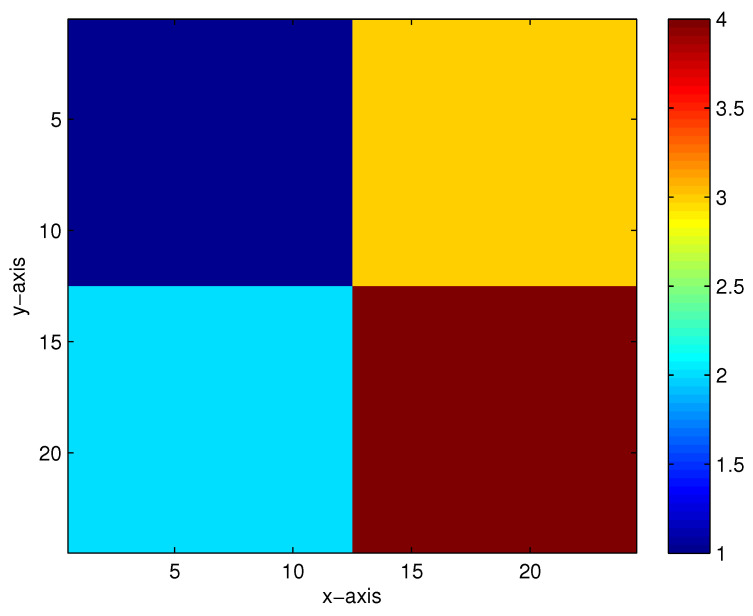
The first true model.

**Figure 3:**
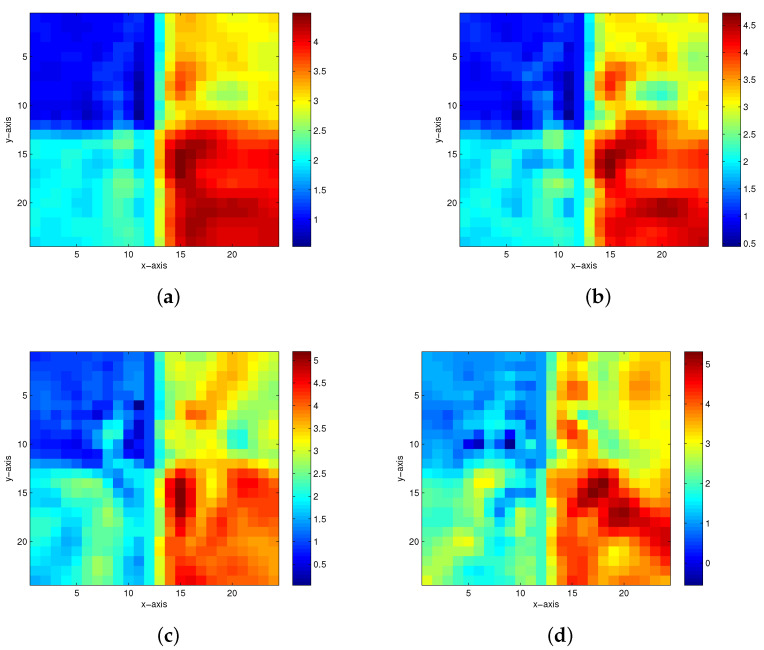
The inversion results of the homotopy method with constraints in the first model. (**a**) 5% Gaussian noise added. (**b**) 10% Gaussian noise added. (**c**) 15% Gaussian noise added. (**d**) 20% Gaussian noise added.

**Figure 4:**
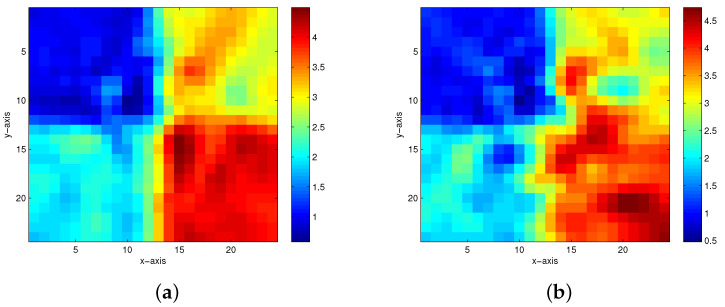
The inversion results of the homotopy method without constraints in the first model. (**a**) 5% Gaussian noise added. (**b**) 10% Gaussian noise added.

The first true model is displayed in Figure 2. When there are 5%, 10%, 15% and 20% Gaussian noises in the measurement data, the inversion results of the homotopy method with constraints and the homotopy method without constraints are shown in Figure 3 and Figure 4, respectively. When the noise level reaches 15% or 20%, the inversion result is still satisfactory for the homotopy method with constraints (see Figure 3c,d); however, the homotopy method without constraints is not convergent.

**Figure 5:**
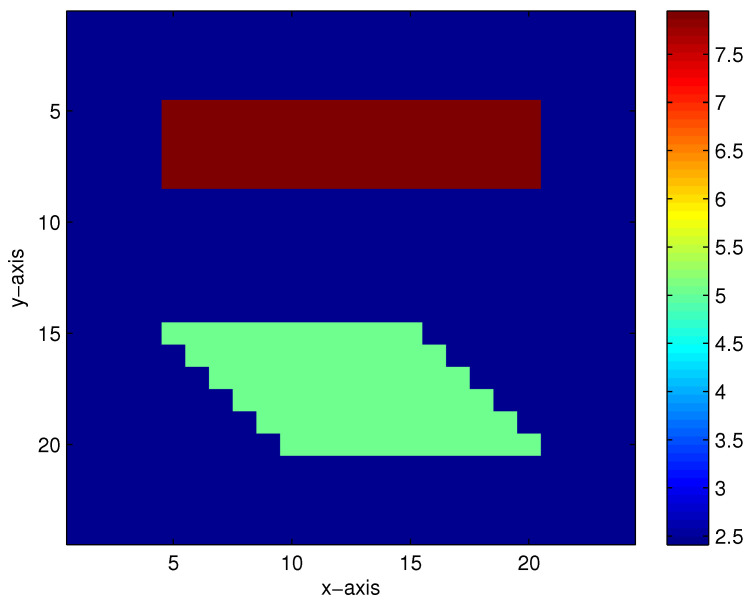
The second true model.

**Figure 6:**
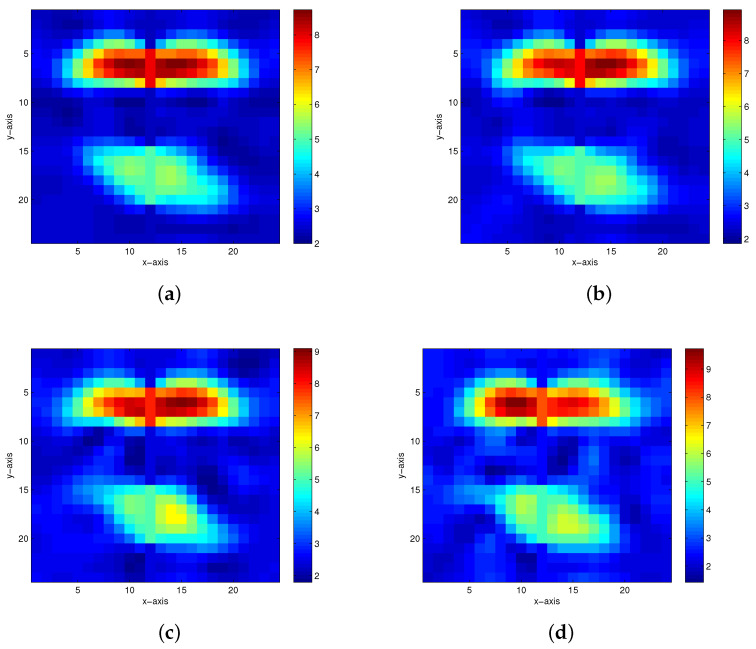
The inversion results of the homotopy method with constraints in the second model. (**a**) 5% Gaussian noise added. (**b**) 10% Gaussian noise added. (**c**) 15% Gaussian noise added. (**d**) 20% Gaussian noise added.

**Figure 7:**
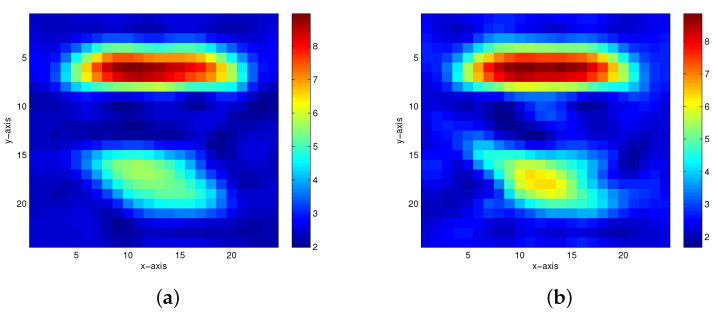
The inversion results of the homotopy method without constraints in the second model. (**a**) 5% Gaussian noise added. (**b**) 10% Gaussian noise added.

The second true model is the model of two anomalous bodies in a homogeneous medium, as shown in Figure 5. Figure 6 and Figure 7 show the inversion results of the homotopy method with constraints and the homotopy method without constraints, respectively, with the noisy data at 5%, 10%, 15% and 20%. Figure 6c,d show the inversion results of the homotopy method with constraints when 15% and 20% Gaussian noises are added to the measurement data, respectively. If the same noise level is used, the homotopy method without constraints is divergent.

For comparison, we investigate three methods for the above two models: the homotopy method with constraints (Equations (12) and (16)), homotopy method without constraints (Equations (17) and (18)), and iterative method with constraints (Equation (12)). The relative errors of inversion results are tabulated in Table 1. Table 1 shows that with 5% and 10% Gaussian noises added, the inversion results by the homotopy method with constraints and the homotopy method without constraints both have very high accuracy; with 15% and 20% Gaussian noises added, the inversion results for the homotopy method with constraints are still satisfactory, but the inversion results for the homotopy method without constraints are not acceptable, which shows the necessity of the constraints; moreover, starting from the same initial estimate, the iteration of the iterative method with constraints is not convergent, which shows the necessity of introducing the homotopy strategy.

To compare the proposed method with other methods (wavelet multiscale method, nonlinear multigrid method, adaptive multigrid conjugate gradient method, homotopy perturbation method), we use all these methods for the first model with 5% Gaussian noise added. When the initial estimate $P^{0}\equiv 5$, the relative errors and CPU run times of these methods are listed in Table 2; When the initial estimate $P^{0}\equiv 1$, the relative errors and CPU run times of these methods are listed in Table 3. From Table 1, Table 2 and Table 3, it is easy to see the basic information about the advantages and disadvantages of the homotopy method with constraints compared to other methods, as presented in Table 4.

In order to study the sensibility of the inversion results on the parameters α, β, $P^{0}$, we use the homotopy method with constraints for the first model with 15% Gaussian noise added, test several values of α, β, $P^{0}$ and list the relative errors in Table 5. From Table 5, it can be seen that the performance of the proposed method is highly sensitive to the values of α and β and insensitive to the value of $P^{0}$. Usually, the optimal regularization parameter is unknown and must be determined by methods such as trial-and-error, cross-validation, L-curve criterion, the discrepancy principle and others. In this paper, we determine the parameters α and β by trial-and-error, for the sake of simplicity. From Table 5, we find that the constraint parameter $\alpha =10^{4}$ performs best, that the regularization parameter $\beta =10^{-5}$ performs best and that the value of initial estimate $P^{0}$ does not affect the inversion result; thus, we let $\alpha =10^{4}$, $\beta =10^{-5}$. It is because of the global convergence property of the homotopy method that the value of initial estimate $P^{0}$ can arbitrarily be chosen. In fact, the value of $P^{0}$ is chosen from range of 0 to 10, since the permeability values are from range of 0 to 10 in reality. The value of $P^{0}$ is usually chosen to be the mean value 5 of 0 to 10, when no prior knowledge is available about the permeability parameter.

**Figure 8:**
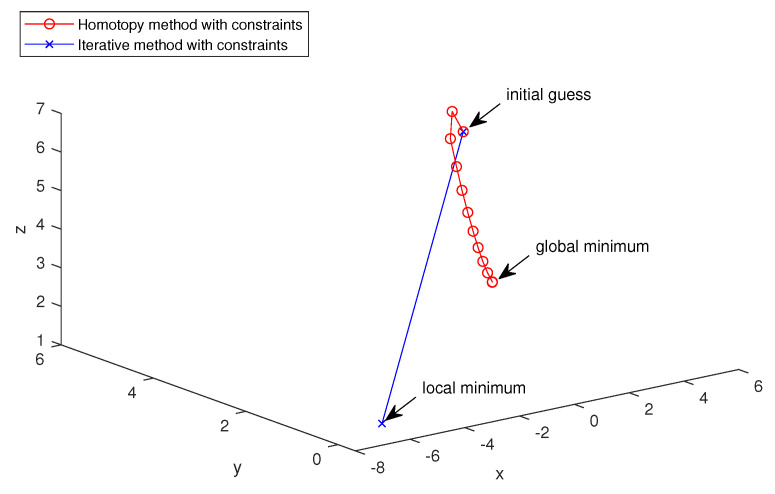
Three-dimensional iterative processes of the homotopy method with constraints and iterative method with constraints.

In order to pictorially demonstrate how the homotopy method avoids the local minimum, we use the homotopy method with constraints and iterative method with constraints for the first model with no noise. Three locations $\left(\frac{4}{24},\frac{4}{24}\right)$, $\left(\frac{4}{24},\frac{20}{24}\right)$, $\left(\frac{20}{24},\frac{4}{24}\right)$ of the parameter model are selected as the (x,y,z) coordinates of three-dimensional plotting. The true parameter values of these locations are 1, 2 and 3, respectively, so the global minimum is (1,2,3). $P^{0}\equiv 5$, so the initial guess is (5,5,5). Figure 8 pictorially shows how the homotopy method with constraints converges to the global minimum from the initial guess (5,5,5) and how the iterative method with constraints converges to the local minimum from the initial guess (5,5,5).

**Figure 9:**
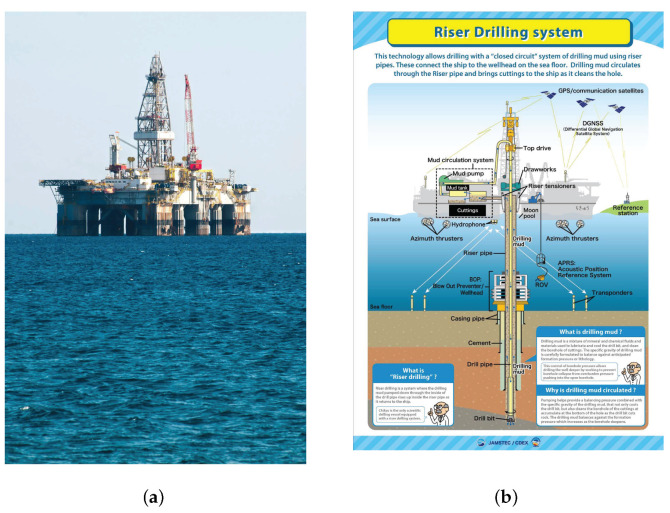
Submarine oil exploration. (**a**) Offshore oil operation platform. (**b**) Riser drilling system.

Submarine oil exploration is one of practical applications of the proposed solution. When reservoir workers value a block and plan to extract oil from the ocean floor, they first need to use methods such as seismic wave inversion and geophysical exploration to show where the oil and gas reservoirs may be. After choosing a location for oil extraction, they starts drilling wells, as shown in Figure 9. Well log data from the drilled well can be used as the constraint data. Based on the complementary characteristics of seismic data and logging data, the joint inversion technology of seismic data and logging data is studied. The regularization homotopy method proposed in this paper is combined with logging constraints to jointly invert the permeability parameters of the nonlinear convection–diffusion equation to obtain the complete distribution information of permeability parameters and further improve the inversion accuracy and anti-noise ability.

## 6. Conclusions

A regularization homotopy strategy is designed for the constrained parameter inversion of partial differential equations. As a practical application, this method has been used successfully to solve the constrained coefficient inversion of the saturation equation in the fractional flow formulation of the two-phase porous media flow equations. It turns out that the homotopy strategy can effectively widen the convergence region of the ordinary numerical methods, and the constraints can effectively improve the noise suppression ability of the inversion methods. Future work will extend this new strategy to the ill-posed image processing problems and the optimal control problems with nonlinear dynamical systems as constraint conditions.

## Figures and Tables

**Table 1:** Relative errors of inversion results by three different methods.

Model Number	Method	5% Noise Level	10% Noise Level	15% Noise Level	20% Noise Level
1	Homotopy method with constraints	8.33%	8.62%	9.24%	9.26%
	Homotopy method without constraints	8.65%	9.06%	×	×
	Iterative method with constraints	×	×	×	×
2	Homotopy method with constraints	17.23%	18.38%	18.72%	18.76%
	Homotopy method without constraints	18.90%	18.93%	×	×
	Iterative method with constraints	×	×	×	×

**Table 2:** Comparison of the proposed method and other methods with $P^{0}\equiv 5$ and 5% Gaussian noise added in the first model.

Method	Relative Error	CPU Run Time (s)
Homotopy method with constraints	8.33%	1335.556
Wavelet multiscale method	×	×
Nonlinear multigrid method	×	×
Adaptive multigrid conjugate gradient method	×	×
Homotopy perturbation method	10.26%	1666.718

**Table 3:** Comparison of the proposed method and other methods with $P^{0}\equiv 1$ and 5% Gaussian noise added in the first model.

Method	Relative Error	CPU Run Time (s)
Homotopy method with constraints	8.35%	1328.798
Wavelet multiscale method	9.39%	952.2021
Nonlinear multigrid method	9.43%	894.9691
Adaptive multigrid conjugate gradient method	9.76%	1072.533
Homotopy perturbation method	10.54%	1674.663

**Table 4:** Advantages and disadvantages of the homotopy method with constraints compared to other methods.

Compared Method	Advantage	Disadvantage
Homotopy method without constraints	better anti-noise ability	×
Iterative method with constraints	larger convergence region	×
Wavelet multiscale method	better anti-noise ability and larger convergence region	more computation
Nonlinear multigrid method	better anti-noise ability and larger convergence region	more computation
Adaptive multigrid conjugate gradient method	better anti-noise ability and larger convergence region	more computation
Homotopy perturbation method	better anti-noise ability	×

**Table 5:** Relative errors of inversion results by the homotopy method with constraints for several values of α, β, $P^{0}$ with 15% Gaussian noise added in the first model.

α with $\beta =10^{-5}$, $P^{0}\equiv 5$	$10^{-2}$	1	$10^{2}$	$10^{4}$
Relative error	×	10.05%	9.26%	9.24%
β with $\alpha =10^{4}$, $P^{0}\equiv 5$	$10^{-3}$	$10^{-4}$	$10^{-5}$	$10^{-6}$
Relative error	14.27%	12.13%	9.24%	×
$P^{0}$ with $\alpha =10^{4}$, $\beta =10^{-5}$	1	3	5	7
Relative error	9.25%	9.23%	9.24%	9.25%
